# Correlates of Eccentric Metrics and Sprint Acceleration and Deceleration Performance in University Athletes

**DOI:** 10.3390/jfmk11020155

**Published:** 2026-04-15

**Authors:** Gregory Gordon, Taygan Nadar, Andrew Green

**Affiliations:** Department of Sport and Movement Studies, Faculty of Health Sciences, University of Johannesburg, Johannesburg 2028, South Africa

**Keywords:** eccentric, sprint, deceleration, jumps, hamstring strength

## Abstract

**Background**: Sprint performance, including acceleration, maximal velocity and deceleration, is crucial for athletic success in field and court-based sports; however, deceleration remains understudied despite its role in change of direction (COD) and match performance. **Methods**: This study addressed this gap by comparing eccentric metrics from countermovement jumps (CMJ), drop jumps (DJ) and the Nordic hamstring exercise (NHE) to 30 m sprint and deceleration ability in 28 university athletes (Age: 20 ± 1 years; Mass: 68 ± 9 kg; Height:166 ± 6 cm). Correlations were analysed with Pearson’s r for normal data and Spearman’s r for non-normal data. **Results**: Significant negative correlations were found between the CMJ and DJ heights and the modified reactive strength index (RSI_MOD_), as well as the reactive strength index (RSI) with sprint time (r = −0.54 to −0.83, *p* < 0.05), while positive correlations were obtained with sprint velocity (r = 0.57 to 0.83, *p* < 0.05). The eccentric mean forces from CMJs and DJs were positively correlated with sprint time and deceleration momentum (r = 0.62 to 0.84, *p* < 0.05). However, there were no significant correlations between NHE eccentric force and any sprint or deceleration metrics. The CMJ and DJ heights, RSI and eccentric mean forces strongly predicted sprint time, velocity, and momentum, but not deceleration performance, highlighting the role of explosive power and reactive strength. The NHE eccentric force had no significant relationships with sprint or deceleration metrics. **Conclusions**: These results highlight that CMJ and DJ are effective predictors of sprint performance, while deceleration efficiency may rely on other biomechanical factors.

## 1. Introduction

Sprint performance, encompassing acceleration, maximal velocity and deceleration, is critical for athletic success in field- and court-based sports [1,2]. However, deceleration, i.e., the ability to rapidly reduce velocity, remains underexplored despite its critical role in executing change of direction (COD) manoeuvres [3,4,5]. Deceleration imposes high mechanical demands, requiring substantial eccentric strength to absorb ground reaction forces (GRFs) of up to 3–6 times body weight, particularly in the quadriceps and hamstrings, which control knee extension and flexion during braking [6,7]. To achieve efficient deceleration with high braking forces, athletes must leverage their balance during the movement, eccentric strength, power and reactive strength, particularly through greater lower limb eccentric knee extensor strength [4,8]. A practical link between these aspects lies in the stretch–shortening cycle (SSC), which underpins both rapid acceleration and effective deceleration, facilitating quick force production to increase velocity during acceleration and aiding in force absorption and control to reduce velocity efficiently during deceleration [9,10].

The countermovement jump (CMJ) is an important test for assessing lower-body power and the SSC, which is critical for athletic performance by enhancing force production and eccentric–concentric transitions [11,12,13]. Within this movement, the eccentric phase is particularly critical, as it facilitates the storage of elastic energy and force absorption—mechanical requirements that directly mirror the demands of elite sprinting [14,15]. High-velocity sprinting relies on the ability to manage substantial GRF during ground contact; consequently, eccentric metrics such as mean power and peak force are strong predictors of acceleration and maximal velocity, with CMJ height specifically demonstrating significant negative correlations with 30 m sprint times (r = −0.71) [15,16]. Beyond linear speed, these eccentric capabilities are essential for deceleration, a mechanically demanding task [17]. This requires the athlete’s quadriceps and hamstrings to lengthen under the load to control the centre of mass displacement [17]. Research by Harper [4] indicates that CMJ eccentric peak force can effectively differentiate between high- and low-performing decelerators, suggesting that superior eccentric strength enhances braking efficiency and neuromuscular control. By capturing variables such as mean force and peak velocity, the CMJ provides a comprehensive profile of an athlete’s ability to absorb and redirect force, making it an indispensable diagnostic tool for evaluating the multifaceted components of multidirectional performance [6,11].

A key test for assessing reactive strength and the SSC, the drop jump (DJ), complements the CMJ by emphasising rapid force absorption upon landing. This is followed by an explosive jump, mimicking the high impact demands of sprinting and deceleration while evaluating neuromuscular readiness for rapid directional changes [4,16,18]. Previous studies have shown that the DJ RSI and jump height are negatively correlated with 30 and 60 m sprint times (r = −0.71) and positively correlated with velocity, reflecting the ability to manage high GRFs within short contact times (<200 ms), mirroring sprinting’s demands [4,6,15,18]. Furthermore, the DJ’s eccentric phase is a critical indicator of deceleration performance, as its mechanics closely resemble braking, indicating enhanced braking efficiency through coordinated quadriceps and hamstring actions [4,17]. Because higher running speeds intensify deceleration demands, similar to greater drop heights increasing eccentric loading, these metrics underscore the DJ’s role in predicting improved deceleration performance, guiding targeted training to optimise force absorption and control [19,20]. Previous studies have shown that a higher DJ RSI is strongly associated with superior horizontal deceleration ability, with DJ eccentric metrics correlating with shorter braking times and enhanced force absorption, highlighting the DJ’s relevance for targeted training to improve braking efficiency in university athletes [4,6].

The Nordic hamstring exercise (NHE) is a primary intervention for isolating eccentric hamstring strength, utilised predominantly for injury risk reduction and the control of knee flexion during high-velocity locomotive tasks [21,22,23]. While the eccentric capacity of the hamstrings facilitates the management of GRFs to maintain sprint momentum, its direct transfer to braking efficiency appears limited [17,24]. Previous research suggests only moderate correlations (r = −0.342 to −0.400) between NHE strength and COD speed [24,25]. Given that deceleration is a task-specific manifestation of multidirectional ability, characterized by a high reliance on quadriceps-dominant eccentric braking, the value of the NHE may be primarily situated within an injury prevention framework rather than as a primary determinant of deceleration magnitude [17].

This study aims to compare eccentric metrics from the CMJ, DJ and NHE to sprint and deceleration performance in university athletes. The objectives were to: (1) examine correlations between CMJ and DJ eccentric metrics with sprint and deceleration outcomes, and (2) assess the relationship between NHE eccentric force and sprint and deceleration outcomes. By clarifying these relationships, this study aimed to bridge a critical research gap and provide practical information for coaches and athletes, enabling them to optimise training programs, enhance performance, and reduce injury risk in sport environments.

## 2. Materials and Methods

### 2.1. Research Design

A cross-sectional correlational research design was implemented to assess the relationships between acceleration and deceleration metrics and eccentric variables.

### 2.2. Participants

A sample of 28 athletes (Age: 20 ± 1 years; Mass: 68 ± 9 kg; Height: 166 ± 6 cm) was recruited from various sporting codes. The athletes in this study were all university student athletes whom all competed for their appropriate teams in competition for the university for a minimum of 3 years. Informed consent was obtained prior to the testing of all participants. Athletes that were selected from the university squads were not injured at the time of testing and those who did not complete all tests were excluded from this study. Institutional ethical approval was granted and obtained for the research project (REC-1359-2022; approval date: 26 January 2022). Calculations performed in G*Power version 3.1.9.7 indicate that a sample size of 28 participants provided approximately 80% power to detect a large effect size (~0.50).

### 2.3. Data Collection

The testing was performed during the in-season of the athletes’ competition schedule. The participants included athletes from two sporting codes, namely hockey and rugby, and involved both male and female participants. Data were collected over 2 days separated by a minimum of 48 h, with athletes informed to refrain from strenuous activity. Testing occurred under similar environmental conditions, with athletes wearing their sport-specific footwear to replicate game-like scenarios. Each participant completed a standardised warm-up prior to testing to optimise performance readiness. On the first day, linear sprint tests and deceleration assessments were performed. Day two involved gym-based assessments: jump tests and Nordic curls.

### 2.4. Linear Maximal Sprint

Sprint times were recorded over 15 and 30 m distances using a Stalker ATS II radar-gun (Stalker Pro, Radar Sales Inc., Minneapolis, MN, USA). The initial 0–15 m of the sprint was considered Phase 1, while the 15–30 m stage was considered Phase 2. Times were recorded to the nearest 0.01 s. Each sprint began from a stationary split stance position with the front foot positioned 30 cm behind the timing gate to prevent a false trigger. Participants were instructed to initiate their own start with no backward step or ‘rocking motion’ and to sprint as fast as possible. Each participant was allowed three trials interspersed by a passive recovery period of at least 2 min. The best 30 m split was used as a ‘criterion’ time in the linear maximal sprint test.

### 2.5. Maximal Deceleration Test

The maximal deceleration ability test was performed with a 15 m maximal sprint followed by a deceleration. The athletes began this test in the same manner as the maximal sprint test, while the Stalker ATS Radar gun was also used to assess the athletes during this test. The participants were instructed to sprint maximally for 15 m followed by an immediate deceleration once they reached the 15 m mark. Once their deceleration led to a complete stop, they were instructed to begin backpedalling back to the 15 m mark to ensure there was a clear moment to signal the end of the deceleration phase. Additionally, if the 15 m sprint time achieved was 5% greater than any of the maximal linear sprint test times the trial was deemed an unsuccessful trial. There were rest periods of 3 min between each trial for the participant [26].

### 2.6. Acceleration and Momentum

The athletes’ acceleration, calculated as velocity divided by time, was determined to assess their change in distance over time, reflecting their sprinting ability after reaching the 15 m mark, while their deceleration was calculated as a negative acceleration value to indicate their stopping efficiency from that point. Momentum was calculated as the product of an athlete’s velocity during multidirectional sprints and their mass.

### 2.7. Multidirectional Sprint Area (MDSA)

The MDSA method quantifies an athlete’s multidirectional sprint ability by measuring velocity across eight directional tests—linear sprint, acceleration−deceleration assessment (ADA), and COD tasks at 45°, 90°, and 135° in both directions—recorded at the 15 and 30 m marks, with deceleration also noting the complete stop point, and plotted as x and y coordinates on a radar graph to form a polygon whose area represents the overall sprint capacity. Velocity, calculated as distance divided by time, was segmented into forward, backward, left, and right views, providing a holistic framework for evaluating and comparing athletes’ multidirectional sprint capabilities [27]. This study only included the forward and backward area segments of the MDSA method. Moreover, this MDSA data has been shown to be valid and reliable from previous research [27].

### 2.8. Jump Tests

During the CMJ, subjects were instructed to stand on VALD Force Decks (Vald Performance, Newstead, Australia) plates and to keep their hands on their hips during the test. Their feet were shoulder-width apart during the jump. The participants were instructed to stand motionless on the force plates for 2–3 s before jumping. Additionally, they were instructed to keep their chest up, with their hands fixed on their hips while looking forward, followed by squatting to a depth where knee flexion was approximately 90° and jumping as high as possible [28]. Similarly, participants performed a DJ test, where they were instructed to drop from a 40 cm platform onto the force plates and once landed immediately jump as high as possible, and then land back onto the force plates [29]. All jump attempts were performed three times, separated by 45 s of passive recovery, and the best jump performed was used for the subsequent analysis [30]. Verbal cues were given to the participants during the jumps to ensure their best performance was achieved.

### 2.9. Nordic Curl

Eccentric hamstring strength was assessed using the VALD NordBord (Vald Performance, Newstead, Australia) [31]. In the present study, participants knelt on a padded board with their ankles secured using the individual ankle attachment, bracing from the lateral malleolus to the medial malleolus at the posterior side of the ankle. Participants performed one set of three maximal NHE repetitions with 30 s breaks between each repetition. The athlete was instructed to gradually lean forward at the slowest possible speed while maximally resisting the movement with both limbs, maintaining a neutral trunk and hip position, in addition to keeping their hands across their chest. Strong verbal reinforcement was provided to encourage maximal effort by the athlete. A valid trial required the force output to reach a distinct peak, indicating maximal eccentric force production, followed by a rapid decrease in force when the participant could no longer counteract gravitational forces [31].

### 2.10. Statistical Analysis

Shapiro–Wilk tests were used to assess data normality and confirm a parametric or non-parametric distribution between variables. Consequently, data were reported as means for the participants. A correlation analysis was made between all variables to fully understand the full extent of the relationships between the different metrics. All analyses were conducted using IBM SPSS Version 30. The significance level was set at *p* < 0.05. Reliability analysis was conducted on the data obtained revealing an average measure of ICC = 0.77, while the coefficient of variations between sprints were 8.81–9.81%. This analysis showed that the data retrieved and used was reliable. Additionally, the VALD ForceDecks used in this study have been found to be a valid and reliable tool with a standardised procedure [32].

## 3. Results

Summary data for sprint time, velocity, acceleration and deceleration are reported in Table 1. Additionally, the kinetics from CMJs, DJs and the NHEs are reported in Table 2. The mean deceleration distance to stop was 3.43 ± 2.14 m. Additionally, the mean of the forward segment of the MDSA for the athletes was 63.57 ± 16.52, whereas the mean of the backward segment was 29.08 ± 8.81.

No significant relationships were observed between eccentric mean force for CMJ, DJ and deceleration time (Phase 1 and Phase 2) (Table 3 and Table 4). The CMJ eccentric mean force had strong positive correlations with Phase 1 sprint momentum (r = 0.76, *p* < 0.05), Phase 1 deceleration momentum (r = 0.80, *p* < 0.05), Phase 2 sprint momentum (r = 0.84, *p* < 0.05) and Phase 2 deceleration momentum (r = 0.47, *p* < 0.05). Similarly, DJ eccentric mean force exhibited strong positive correlations with Phase 1 sprint momentum (r = 0.621, *p* < 0.05), Phase 1 deceleration momentum (r = 0.73, *p* < 0.05), Phase 2 sprint momentum (r = 0.75, *p* < 0.05), and Phase 2 deceleration momentum (r = 0.53, *p* < 0.05).

The CMJ height exhibited significant negative correlations with the Phase 1 deceleration time (r = −0.43, *p* < 0.05), Phase 1 sprint time (r = −0.57, *p* < 0.05), Phase 2 sprint time (r = −0.83, *p* < 0.05), and significant positive correlations with Phase 1 sprint velocity (r = 0.59, *p* < 0.05), Phase 2 sprint velocity (r = 0.83, *p* < 0.05), and acceleration (Phase 1 sprint acceleration: r = 0.58, *p* < 0.05; Phase 2 sprint acceleration: r = 0.83, *p* < 0.05). No significant correlations were found with deceleration velocity (Table 3). The DJ height was significantly negatively correlated with Phase 1 sprint time (r = −0.55, *p* < 0.05), Phase 2 sprint time (r = −0.72, *p* < 0.05), and positively correlated with Phase 1 sprint velocity (r = 0.57, *p* < 0.05), Phase 2 sprint velocity (r = 0.72, *p* < 0.05), and acceleration (Phase 1 Sprint Acceleration: r = 0.55, *p* < 0.05; Phase 2 sprint acceleration: r = 0.72, *p* < 0.05). No significant correlations were found with deceleration time (Table 4).

The CMJ modified reactive strength index-modified (RSI_MOD_) was significantly negatively correlated with Phase 1 deceleration time (r = −0.39, *p* < 0.05), indicating that athletes with higher reactive strength stopped faster in the initial deceleration phase. No significant correlation was observed with Phase 2 deceleration time (Table 3). For sprint performance, CMJ RSI_MOD_ exhibited strong negative correlations with both Phase 1 (r = −0.68, *p* < 0.05) and Phase 2 (r = −0.73, *p* < 0.05) sprint times, suggesting that higher reactive strength predicts faster sprinting across both phases. Regarding velocity, CMJ RSI_MOD_ was strongly positively correlated with the Phase 1 (r = 0.54, *p* < 0.05) and Phase 2 (r = 0.73, *p* < 0.05) sprint velocities, indicating that a higher reactive strength is associated with a greater sprint speed. No significant correlations were found with Phase 1 or Phase 2 deceleration velocities (Table 3).

The DJ RSI was significantly negatively correlated with Phase 1 deceleration time (r = −0.38, *p* < 0.05), suggesting that athletes with a higher DJ reactive strength achieved faster initial braking. No significant correlation was observed with Phase 2 deceleration time (Table 4). For sprint performance, the DJ RSI exhibited a strong negative correlation with Phase 2 sprint time (r = −0.51, *p* < 0.05), but no significant correlation with Phase 1 sprint time (r = −0.36, *p* = 0.06), indicating that DJ RSI primarily predicts faster sprinting in the later phase. For velocities, DJ RSI was significantly positively correlated with the Phase 1 (r = 0.38, *p* < 0.05) and Phase 2 (r = 0.51, *p* < 0.05) sprint velocities, suggesting that higher reactive strength enhances sprint speeds, particularly in the later phase. No significant correlations were found with the Phase 1 or Phase 2 deceleration velocities (Table 4).

No significant differences were reported between Nordic curl forces and sprint and deceleration metrics (Table 3 and Table 4).

## 4. Discussion

The primary aim of this study was to investigate the relationships between eccentric kinetics obtained from CMJs, DJs and the NHE and both sprint acceleration and deceleration performance. The results demonstrated significant relationships for CMJ and DJ heights, RSI and eccentric mean forces with sprint time, velocity and momentum. However, no statistically significant relationships were reported for NHE eccentric force and deceleration metrics, and there were non-significant relationships for CMJ and DJ eccentric forces with sprint and deceleration times and velocity, which contrasts with some previous findings, particularly those involving professional athletes.

Previous studies have indicated a relationship between jump height and sprint performance [4,6,11,15,16,17]. Specifically, it was expected that greater CMJ and DJ heights would be negatively correlated with sprint time and positively correlated with sprint velocity because greater explosive power enhances sprint performance [4,15,17,33,34,35]. The data confirmed these expectations, with CMJ height exhibiting strong negative correlations with the 0–15 and 15–30 m sprint times and positive correlations with the corresponding sprint velocities. Similarly, DJ height exhibited robust negative correlations with sprint time and positive correlations with velocity, indicating that a larger jump height results in a shorter sprint time. These findings align closely with previous research that reported a significant negative correlation between vertical jump height and 30 m sprint time (r = −0.71) in young male elite sprinters [15] as well as in amateur and semi-professional football players [36]. Furthermore, sprinters with larger vertical jumps were found to have faster sprinting times [37]. The consistency between CMJs and DJs suggests that both tests effectively capture lower-body power, with CMJ countermovement and DJ reactive strength contributing to sprint acceleration and speed maintenance [4,17].

The CMJ RSI_MOD_ and DJ RSI were anticipated to negatively correlate with sprint time and positively correlate with sprint velocity, reinforcing the role of the SSC in rapid force production [11,12,38]. The current study supported these expectations, with CMJ RSI_MOD_ exhibiting strong negative correlations with Phase 1 and Phase 2 sprint times and positive correlations with the corresponding sprint velocities. In contrast, the DJ RSI demonstrated a significant negative correlation with the 15–30 m sprint time and positive correlations with velocity. These results were consistent with those of McLellan [12], who found that the RSI predicts sprint performance in athletes, as well as identifying its role in velocity generation [39]. The stronger correlations in the later sprint phases (15–30 m) suggest that reactive strength is particularly crucial for maintaining speed [15,36]. This aligns with biomechanical reasoning and previous research showing that a higher RSI ability, which reflects the capability to rapidly switch from an eccentric to a concentric action, facilitates shorter ground-contact times and higher sprinting velocities [4,40].

Furthermore, deceleration times were expected to negatively correlate with CMJ height, CMJ RSI_MOD_ and DJ RSI because explosive power and reactive strength should facilitate faster braking during deceleration [17]. The findings of the current study partially met these expectations, with significant negative correlations for Phase 1 deceleration time but no significant relationships for Phase 2 deceleration time or velocity. These findings align with previous research that found CMJ concentric and eccentric peak forces differentiate high decelerators among university athletes, suggesting that the CMJ’s countermovement mimics eccentric loading during the initial braking of the sprint [4]. However, they also found that concentric forces were the key discriminators of deceleration, whereas we emphasised momentum and reactive qualities [4]. There are several likely reasons why concentric actions may assist with horizontal deceleration, including the ability of concentric muscular contractions to develop force more rapidly than eccentric contractions [41]. Additionally, the capacity to generate high concentric forces at elevated joint angular velocities is linked to increased thickness and pennation angle of the leg extensor muscles, which contribute to enhanced isometric force, rate of force development (RFD) and eccentric leg stiffness [42,43]. The lack of significant correlations for the later deceleration phases and velocities may reflect the complex biomechanics of deceleration, where quadriceps strength, technique and fatigue play larger roles [17]. However, the non-significant DJ height correlations with deceleration time contrast with expectations, and are possibly because the DJ’s reactive strength is less relevant for braking than the CMJ countermovement, highlighting test-specific differences [44].

In addition, the CMJ and DJ eccentric mean forces were expected to negatively correlate with sprint and deceleration times and positively correlate with velocity, as stronger eccentric forces enhance force absorption [4]. Contrary to expectations, no significant correlations were found, which differs from previous research that linked eccentric force to jump performance in athletes [45]. Additionally, the 15 m to stop task may not fully engage the eccentric force, as braking may rely more on technique or other muscle groups [4]. Our research found that eccentric mean force from jumps is more relatable to momentum than other sprinting metrics.

Because eccentric muscle actions are largely responsible for deceleration and the function of mass, it was anticipated that positive correlations would be reported between eccentric jump kinetics and deceleration momentum [4]. This study confirmed these expectations, with strong positive correlations across all momentum metrics for both CMJ and DJ eccentric mean forces. This emphasises the role of the athlete’s force in momentum generation and is consistent with previous research that linked eccentric force to jump performance in athletes [45]. The similarity with these studies, conducted across various athlete levels, suggests that the contribution of eccentric strength to momentum is a universal biomechanical principle [5]. The strong relationship between eccentric force and momentum likely exposes the mechanics of braking, in which an athlete with more momentum entering a deceleration must absorb greater force, and those with higher eccentric capacities are better able to handle that load. Additionally, athletes who can apply greater posterior braking forces (and thus high eccentric strength) before a COD show superior deceleration ability [46]. Furthermore, our study found that athletes with greater eccentric jump forces also had higher sprint momentum. This reinforces the rationale that strength training targeting eccentric force is critical for maximising momentum during sprinting and deceleration tasks. Moreover, this strong correlation between momentum and sprinting, as well as deceleration tasks, suggests that eccentric strength may assist athletes in handling larger kinetic loads and thus contribute to managing their momentum.

The NHE eccentric force was expected to negatively correlate with deceleration time and positively correlate with velocity, momentum, and force–time curve area, as stronger hamstrings enhance braking and force production [22,24]. However, no significant correlations were observed across these metrics, contrasting with previous research, which found that NHE strength improved sprint deceleration [22,47]. The lack of significant correlations between NHE eccentric force and deceleration metrics in our study may be attributed to the task-specific emphasis on quadriceps eccentric function. This was highlighted by Harper [17], who found that the quadriceps were primarily responsible for absorbing impact forces during the initial phase of horizontal deceleration, whereas the hamstrings play a secondary role in controlling knee flexion and providing stability. This biomechanical insight suggests that the 15 m to stop task prioritises quadriceps-dominated force absorption, similar to the eccentric phases of CMJ and DJ, which engage both the quadriceps and hamstrings, but differs from the NHE, which isolates hamstring eccentric strength. This potentially explains the observed discrepancies with previous studies involving dynamic hamstring assessments [22,25]. Additionally, the quadriceps’ dominance in force production may limit the NHE’s direct impact on sprint times compared to the CMJ or DJ metrics [17,25].

### 4.1. Implications for Training and Future Research

The strong correlations among CMJ and DJ heights, RSI and sprint performance reinforce the value of plyometric and jump-focused training in university athletes to enhance sprint speed. These findings are particularly relevant for coaches working with developing athletes, as they highlight the transferability of explosive power to sprinting tasks. An athlete’s explosive power, developed through plyometric training, may be considered a universal predictor of sprint performance in developing athletes, regardless of the slight variations in training routines. Training interventions targeting SSC efficiency, such as plyometric exercises, can enhance both acceleration and deceleration capabilities in athletes [48]. The positive correlations between eccentric mean force and momentum suggest that strength training targeting eccentric force can maximise momentum generation, which is beneficial for both sprinting and controlled deceleration. However, the non-significant NHE correlations with deceleration metrics indicate that the role of hamstring strength may be task-specific, particularly for university athletes with moderate training ages. Coaches should consider dynamic NHE variations or complementary exercises, such as sprint training, to enhance hamstring contributions to deceleration, as previously suggested in the literature [22]. Future research could also include introducing new tests such as cardiopulmonary exercise testing (CPET) which is seen as a gold standard assessment for endurance testing [49] as well as assessing muscle activations using electromyography (EMG).

### 4.2. Limitations

The inclusion of inertial sensors during sprint tests would provide more detailed kinematic data, allowing for a more comprehensive analysis of the acceleration and deceleration phases [50]. Additionally, incorporating isokinetic testing to control the velocity of eccentric exercises would offer a standardised approach for assessing muscle function across different angular velocities [51]. These enhancements would contribute to a more nuanced understanding of the relationship between eccentric strength and sprint performance. Moreover, the definition of deceleration can be further examined as this task can be defined with different thresholds, such as time or distance. Furthermore, longitudinal studies examining the effects of specific training interventions could determine the causal mechanisms underlying performance improvements and optimise training protocols for athletes in various disciplines. Additionally, the inclusion of a larger sample of participants may also improve the power and effect size thus providing better interpretation of the results.

## 5. Conclusions

This study investigated the relationships among eccentric kinetics from CMJs, DJs and the NHE with sprint acceleration and deceleration in university athletes. Significant correlations were found between CMJ and DJ eccentric metrics, such as jump height and sprint performance, including faster sprint times and higher velocities, aligning with expectations based on previous research. These findings reflect the importance of lower-body power and reactive strength in sprinting. However, contrary to expectations, no significant correlations emerged with deceleration performance. This suggests that while CMJ and DJ metrics are strong predictors of sprint speed, deceleration efficiency may depend on the distinct biomechanical factors not captured by these jump-based assessments. The NHE eccentric force exhibited no significant correlations with either sprint or deceleration outcomes. This differs from the weaker correlations expected from the results in this study and may be due to the quadriceps dominance in the sprint tests. These findings could guide coaches in developing targeted plyometric and strength training programs to enhance sprint speed, improve deceleration efficiency, and reduce injury risk in university athletes, contributing to both performance optimisation and athlete safety. Despite this, the NHE’s established role in hamstring injury prevention remains a key consideration for athlete safety, even if its direct impact on performance metrics is limited in this context.

These findings have practical implications for coaches and athletes. Plyometric training emphasising the CMJ and DJ can effectively enhance sprint speed, while deceleration efficiency may require targeted interventions beyond jump-based metrics, such as dynamic eccentric hamstring exercises. By clarifying these relationships, this study bridges a critical gap, guiding the development of training programs that optimise performance and reduce injury risk in dynamic sports environments.

## Figures and Tables

**Table 1:** Results (means and standard deviations) of sprint and deceleration tests.

	Phase 1	Phase 2
Sprint Test Time (s)	3.09 ± 0.29	2.05 ± 0.22
Deceleration Test Time (s)	3.31 ± 0.22	1.07 ± 0.35
Sprint Test Velocity (m/s)	5.13 ± 1.04	7.40 ± 0.80
Deceleration Test Velocity (m/s)	4.43 ± 0.53	−2.98 ± 0.98
Sprint Test Momentum (m/s·kg)	338.69 ± 95.14	488.71 ± 106.66
Deceleration Test Momentum (m/s·kg)	292.16 ± 59.74	−197.09 ± 74.65
Sprint Test Acceleration (m/s^2^)	1.61 ± 0.34	3.69 ± 0.8
Deceleration Test Acceleration (m/s^2^)	1.39 ± 0.18	−1.78 ± 2.32

**Table 2:** Results (means and standard deviations) of CMJ, DJ, and NHE Tests.

	Mean ± SD
CMJ Eccentric Mean Force [N]	652.96 ± 110.22
CMJ Height [cm]	30.09 ± 8.89
CMJ Eccentric Peak Velocity [m/s]	−0.73 ± 0.27
CMJ RSI_MOD_ [m/s]	0.37 ± 0.15
DJ Height [cm]	29.72 ± 8.02
DJ Eccentric Mean Force [N]	1519.04 ± 398.22
DJ Peak Drive-Off Force [N]	1817.61 ± 486.75
DJ RSI	1.10 ± 0.34
DJ Eccentric Mean Force [N]	1519 ± 398.27
DJ Drop Landing RFD [N/s]	68,496.07 ± 33,832.28
Nordic Max Force (N)	537.39 ± 155.57

**Table 3:** Correlations between sprints and decelerations with countermovement jump kinetics.

	Deceleration	Phase 1								Phase 2								Multidirectional Sprint Area	
	Deceleration Distance Till Stopped	Sprint Time	Deceleration Time	Sprint Velocity	Deceleration Velocity	Sprint Momentum	Deceleration Momentum	Sprint Acceleration	Deceleration	Sprint Time	Deceleration Time	Sprint Velocity	Deceleration Velocity	Sprint Momentum	Deceleration Momentum	Sprint Acceleration	Deceleration	Area Forward	Area Backward
Eccentric Mean Force [N]	0.04	−0.36	−0.25	0.28	0.24	0.28	0.17	0.31	0.27	−0.28	−0.13	0.76 **	0.80 **	0.84 **	0.47 *	0.28	0.16	0.52 **	0.56 **
Jump Height [cm]	0.19	−0.57 **	−0.43 *	0.59 **	0.19	0.825 **	0.23	0.58 **	0.42 *	−0.83 **	−0.04	0.52 **	0.27	0.66 **	0.20	0.83 **	0.12	0.71 **	0.72 **
Eccentric Peak Velocity [m/s]	0.40 *	−0.38 *	−0.02	0.14	0.08	−0.03	0.31	0.23	0.05	0.03	0.31	0.05	0.21	0.13	0.35	−0.03	−0.48 *	0.03	0.03
RSI_MOD_ [m/s]	0.11	−0.68 **	−0.39 *	0.54 **	0.16	0.73 **	0.16	0.54 **	0.41 *	−0.73 **	−0.09	0.47 *	0.45 *	0.71 **	0.32	0.73 **	0.07	0.61 **	0.59 **

** Correlation is significant at the 0.01 level (2-tailed). * Correlation is significant at the 0.05 level (2-tailed).

**Table 4:** Correlations between sprints and decelerations with drop jump kinetics and Nordic curls.

	Deceleration	Phase 1								Phase 2								Multidirectional Sprint Area	
	Deceleration Distance Till Stopped	Sprint Time	Deceleration Time	Sprint Velocity	Deceleration Velocity	Sprint Momentum	Deceleration Momentum	Sprint Acceleration	Deceleration	Sprint Time	Deceleration Time	Sprint Velocity	Deceleration Velocity	Sprint Momentum	Deceleration Momentum	Sprint Acceleration	Deceleration	Area Forward	Area Backward
Jump Height [cm]	0.21	−0.55 **	−0.37	0.57 **	0.17	0.72 **	0.24	0.55 **	0.37	−0.72 **	0.00	0.57 **	0.32	0.60 **	0.32	0.72 **	0.07	0.64 **	0.71 **
Eccentric Mean Force [N]	0.14	−0.19	−0.21	0.13	0.22	0.19	0.25	0.16	0.21	−0.19	0.00	0.62 **	0.73 **	0.75 **	0.53 **	0.19	0.08	0.57 **	0.60 **
Peak Drive-Off Force [N]	0.12	−0.34	−0.16	0.35	0.11	0.46 *	0.23	0.35	0.16	−0.46 *	−0.09	0.66 **	0.71 **	0.78 **	0.47 *	0.46 *	0.17	0.70 **	0.72 **
RSI	0.17	−0.36	−0.38 *	0.38 *	0.18	0.51 **	0.25	0.36	0.38 *	−0.51 **	−0.02	0.54 **	0.43 *	0.64 **	0.40 *	0.51 **	0.02	0.66 **	0.66 **
Eccentric Mean Force [N]	0.14	−0.17	−0.28	0.13	0.22	0.19	0.25	0.16	0.27	−0.19	0.02	0.62 **	−0.08	0.06	0.02	0.19	0.08	0.23	0.20
Drop Landing RFD [N/s]	0.02	−0.11	−0.06	0.09	0.01	−0.08	0.10	0.11	0.07	0.08	−0.02	0.22	0.07	0.07	0.13	−0.08	−0.15	−0.04	0.06
Nordic Max Force (N)	0.05	−0.26	−0.09	0.33	−0.02	0.42 *	0.14	0.34	0.05	−0.42 *	−0.03	0.55 **	0.33	0.56 **	0.26	0.42 *	0.18	0.63 **	0.61 **

** Correlation is significant at the 0.01 level (2-tailed). * Correlation is significant at the 0.05 level (2-tailed).

## Data Availability

The data presented in this study are available on request from the corresponding author.
